# Pathology and Treatment of Yellow Fever; with Some Remarks upon the Nature of Its Cause and Its Prevention

**Published:** 1881-07

**Authors:** H. D. Schmidt

**Affiliations:** New Orleans, La.


### ARTICLE IV.

THE PATHOLOGY AND TREATMENT OF YELLOW FEVER; WITH SOME REMARKS UPON THE NATURE OF ITS CAUSE AND ITS PREVENTION. By H. D. Schmidt, M.D., New Orleans, La. (Continued from page 622, June No., 1881.)

### THE STOMACH.

In all serious cases of yellow fever, whether terminating favorably or not, this organ, like the liver, is deeply involved in the pathological process, originally called forth by the introduction of the specific poison of the disease into the blood. In consequence, the clinical symptoms arising from this implication of the stomach, and consisting mainly in a disturbance of its circulation, are, as we have seen, very annoying to the physician, and, at the same time, so distressing to the patient, as to have induced a number of the older physicians and authors to even regard this organ as the true seat of the whole disease. Although it may be justly presumed that the functional disturbances of the stomach are partly depending on the common cause—the presence of the poison in the blood—it is nevertheless true, that the congestion of the gastric veins is only secondary in its nature, depending upon the imperfect portal circulation in the liver. The condition of the stomach, therefore, may in all cases, as I have before hinted at, serve as a true index to that of the liver. And, in order to render the demonstration of the disturbances in the gastric circulation more perspicuous, it may be proper to briefly review the particular blood vessels supplying the mucous membrane of the stomach with blood, and their arrangement.

It will be remembered that the larger blood vessels sent to the stomach form two arches along the so-called curvatures of this organ. The arterial arch along the lesser curvature is formed by the union of the *coronary* artery and of the *pyloric* branch of the hepatic, while that along the greater curvature is formed by the right *gastro-epiploic* branch of the splenic artery; the latter, also, furnishes some small branches, the *vasa brevia*, to the "cul de sac." The veins, corresponding to these arteries, return their blood to the splenic and superior mesenteric veins, which by their junction form the portal vein ; sometimes the coronary vein joins the latter directly. The branches arising from these vessels, both arteries and veins, in entering the walls of the stomach, penetrate through its muscular coat to be distributed throughout the sub-mucous tissue in which they divide into subordinate branches. Of these, the finest arterial branches penetrate the muscular layer of the mucous membrane, and arriving at the glandular layer, give rise to a network of capillary vessels with oblong meshes surrounding the individual gastric glands, and extending to the vicinity of their apertures. Here the capillaries of the network somewhat enlarge in diameter, while their meshes assume a more regular polygonal form, each mesh surrounding the aperture of a gland. From these larger capillaries the venous radicles take their origin in the form of minute branches, a small number of which, representing a small district of the mucous membrane, join to form a somewhat larger vessel, which, without receiving any additional branches, descends vertically to join a venous network, extending between the glandular and muscular layers of the mucous membrane. From this network, other branches arise, which, by penetrating the muscular layer, join those larger veins ramifying, in company with the arteries, throughout the sub-mucous tissue, and which finally penetrate the other coats of the stomach to reach their ultimate destination, the splenic, or superior mesenteric vein.

As regards the function of the capillaries of the mucous membrane of the stomach, it may be mentioned that the larger kind, forming polygonal meshes around the apertures of the gastric glands, have been regarded as subserving the process of absorption, while those surrounding the gastric glands themselves, smaller in diameter, and forming oblong meshes, are considered to furnish the material for the secretion (*Frey*). We may now proceed with the description of the pathological changes taking place in the mucous membrane of this organ.

In almost all cases of yellow fever, this membrane is found more or less congested. The congestion, however, does not extend uniformly throughout the whole membrane, or even larger portions of it, but is confined to smaller or larger spots or districts, in which it is observed to proceed from one or more centers. From these centers it extends or radiates in a lesser degree, either gradually to be lost, or to pass over to another congested district. It is owing to this peculiarity of the congestion that it presents no uniformity of character, but is observed to spread irregularly over larger or smaller portions of the membrane. Neither are the congested portions of the latter limited to any particular region of the organ; they may be nearer to the cardiac, or to the pyloric extremity; in most instances, however, they are found in that portion forming the greater curvature. In examining the congested portions of the mucous membrane with a loupe, magnifying about five diameters, the congestion will be found confined to the minute venules, presenting an arborescent arrangement resembling the broken meshes of a vascular network (Fig. 6, *a*). From the center of each individual district, corresponding to the apparent trunk of these minute vessels, a few fine branches are seen to proceed, which in their turn give rise to still finer ones, the whole resembling the branch of a tree without leaves. From our description of the arrangement of the blood vessels of the mucous membrane of the stomach, given above, it may be inferred that the finest of these vessels are identical with those minute venules arising in the upper and larger capillaries, surrounding the apertures of the gastric glands, and that the branches, formed by their junction, converge to a common center, in order to form one of those larger vessels, which, without receiving any additional branches, descend betwen the gastric glands (Fig. 5) to join their respective venous network extending between the glandular and muscular layer of the mucous membrane.

This particular form of congestion prevails in a greater or lesser degree in almost all the congested portions of the membrane. Each descending venule or vein, as has been seen, forms one of the centers, while its minute tributaries represent the districts. Nevertheless, in most fatal cases, besides this particular form of congestion, a number of small and defined spots or patches are also found, presenting a distinct red color, and resembling small ecchymoses or extravasations of blood. Their size is very limited, ranging from that of a mere dot to about two or three mm., seldom more; and if they are examined with a strong loupe, they are found to consist of an unbroken network of minute vessels, congested with blood (Fig. 6, *b*), and identical with that network of large capillaries which surrounds the apertures of the gastric glands. In most instances, these small congested spots or patches are found between or along the sides of the plicæ, sometimes extending over the summit.

In examining thin sections made vertically through one of these small congested patches, the peculiar character of the congestion can be clearly demonstrated. In such sections (Fig. 5), then, it will be found that, while the arteries with their *capillaries*, surrounding the gastric glands throughout nearly their entire length, are *empty*, all the veins throughout the entire wall of the congested portion of the stomach are congested with blood. The congestion commences in the network of large capillaries, surrounding the apertures of the glands, whence it extends through the venous radicles to all other veins of the organ, contained in the section. These large capillaries, especially, are distended with blood corpuscles, which, in a number of places, may be observed to have ruptured the minute vessels, and extravasated into the surrounding tissue. In other places, again, no rupture has taken place, but, in consequence of the stasis of blood, which obviously must have existed in these localities, the blood corpuscles have parted with their hæmoglobin, which, passing through the walls of the vessels, was absorbed by the neighboring epithelial cells, as shown by the brown color they present to the eye of the observer. Thin sections, sliced off with a sharp knife from the surface of one of these spots, embracing the outer epithelium, also show distinctly the condition of the membrane just described. It will be easily understood that the hæmorrhagic effusions into the stomach, occurring during the course of yellow fever, and represented by the "black vomit," are derived from the rupture of these minute vessels. In the numerous sections of the mucous membrane of the stomach which I have examined, I have failed to discover any product of inflammation.

It has been asserted, and is believed by a number of physicians, that in yellow fever the epithelium of the stomach underwent fatty degeneration. During the epidemic of 1867, but particularly during that of 1878, I had carefully examined the fresh epithelium of a considerable number of stomachs without finding any traces of this degenerative process. Nor can it be demonstrated in the thin sections of the hardened specimen, in which the details of the cellular elements can be minutely studied. On the contrary, it is not only found that the epithelial and glandular cells contain no fat globules, but, moreover, that their protoplasm is very readily colored by the staining liquid.

With the exception of the pathological changes above described, none are observed in the remaining tissues of the stomach.

### THE INTESTINES.

The distribution and arrangement of the blood-vessels of these organs being very nearly the same as that met with in the stomach, it may be expected that the congestion of these parts also partakes of the same character. But though, in the greatest number of fatal cases, the larger veins, emerging from the walls of the intestines to become tributaries of the portal vein, are found filled with blood, the congestion of the minute vessels of the mucous membrane very rarely attains as high a degree as in the same membrane of the stomach. An explanation of this fact may be found in the distance intervening between the portal vein and the mucous membrane of the intestines, being greater than that existing between this vessel and the mucous membrane of the stomach. The first effect of any retardation of the portal circulation of the liver will, of course, be felt in those tributaries which the portal vein receives nearest to this organ. For this reason, hæmorrhages into the intestines only take place in very severe cases, though a congestion of the venous radicles is frequently observed, especially in the upper portion of the small intestines. When blood is observed to pass from the bowels, however, it does not always signify that a hæmorrhage from the mucous membrane of the intestines has occurred, as it may possibly be derived from a hæmorrhage into the stomach, and, instead of being vomited, be discharged by way of these organs. The appearance which the congested mucous membrane of the intestines presents to the eye of the observer is very similar to that of the congested portions of the stomach above described, having its seat in the minute venous radicles. Those small red spots or patches, formed by a congestion of the larger capillaries, surrounding the apertures of the glands, are rarely met with upon the mucous membrane of the intestines.

### THE SPLEEN.

This organ is almost always found in a normal condition; any deviation from the latter should, as in the case of the lungs, be considered as having no relation with yellow fever, but depending upon other causes. The only pathological phenomenon which the microscopical examination in some cases reveals, consists in the presence of small masses of black pigment, deposited in the pulp of this organ, but which are frequently met with in malarial spleens and livers. The blood-corpuscles, neither, present anything remarkable in their form, or otherwise. These statements are based upon the examination of the fresh pulp in a number of cases, and, furthermore, upon that of thin microscopical sections of the hardened specimen.

### THE KIDNEYS.

In almost all fatal cases of yellow fever, the microscopical examination shows that certain pathological changes have taken place in the parenchyma of these organs. These changes, as I shall show directly, chiefly consist in a degeneration of the epithelium lining the uriniferous tubules, and, in some instances, they exist to so small an extent as not to alter the normal appearances presented by the cut surfaces of the kidneys to the naked eye. Among the autopsies which I performed during the epidemic of 1878, I met with a number of such specimens of kidney, the normal appearance of which so much misled me as to make me indifferent to their preservation. Subsequent microscopical examinations of thin sections made from similar specimens, however, showed me that even in these kidneys of normal appearance the peculiar changes do exist, and are easily demonstrated; and, in consideration of their almost constant occurrence, they may safely be regarded as characteristic of yellow fever.

As the anatomical construction of the kidneys is quite complicated, I do not consider it out of place to briefly review it before proceeding to the description and discussion of the pathological phenomena observed in these organs.

When a kidney is divided longitudinally, the cut surface appears as if consisting of two different substances. The one forming the external portion of the organ, and accordingly called "cortical," presents a granular appearance, while the other, called "medullary," is collected in conical or pyramidal masses, and appears to consist of bundles of fibers, converging in each pyramid from the base to the apex. Thus the kidney may be regarded as composed of the cortical substance and about a dozen, or more, of these conical bodies, the bases of which are connected and surrounded by the former, while their apices, represented by the *renal papillæ*, project into the cavity, or *sinus*, of the organ; and, furthermore, each pyramid, together with its respective portion of cortical substance, may be looked upon as a separate gland. In embryonic life, the kidney really consists of a number of minor kidneys, held together by connective tissue, which subsequently become blended with each other. The secreting portion of the kidney is represented by an immense number of tubules, lined by a layer of epithelial cells, the true secreting elements. The mutual relationship of these tubules is quite simple, consisting in the junction which a number of the primary tubules form with a particular kind of straight or collecting tubules, and in the union of these to form, finally, still larger ones, the *ductus papillares;* but the changes which they undergo in their diameter and in the direction of their course, together with the peculiar arrangement and distribution of the blood-vessels, render the study of the renal parenchyma rather complicated. The apparent difference existing between the cortical and medullary substances is owing to these variations existing in the diameter, form, and course of the uriniferous tubules, and in the arrangement of their minute blood-vessels, though a distinction may be presumed to exist in their functions; that is, the cortical substance representing the proper secreting portion of the gland, while the medullary, consisting entirely of straight and larger tubules, represents that portion through which the secretion is discharged.

If the cut surface of the cortical substance is examined with a sufficiently strong loupe, a considerable number of minute spherical bodies will be observed; these are the so-called "Malpighian bodies," upon which the uriniferous tubules commence. Each of these bodies consists of a conglomeration of minute vessels, surrounded by a thin capsule, the continuation of which, in the form of a short and narrow constriction or neck, represents the commencement of a uriniferous tubule. The capsule itself, consists only of a layer of flat pavement cells (endothelium), surrounded by a thin layer of connective tissue, while the tubule, throughout its entire length, represents a tube of a structureless so-called basement-membrane, lined by an epithelial layer of cells, which, in different portions of the tubule, assume a different form and arrangement. The tubule, after having arisen by its neck from the capsule, rapidly enlarges in diameter to form a comparatively wide tube, which, bending into several convolutions, pursues its course in a slightly oblique direction toward the center of the kidney; but, diminishing again in diameter to about one-third of that of the convoluted portion, it suddenly makes a turn and descends vertically toward the medullary substance, forming thus what is called the *descending limb* of the tubule. After penetrating, to a lesser or greater distance, into the medullary substance, it makes, opposite to the side whence it came, a short bend upon itself (loop of *Henle*), and ascends again in a vertical direction, forming the *ascending limb.* Having passed beyond the height of its respective Malpighian body, it again enlarges in diameter, and forms the *intermediate* portion or canal, consisting of a few convolutions. This portion, after being once more reduced in diameter to a very fine tubule, makes a turn sideward to join its respective *collecting* tubule. The collecting tubules commence near the surface of the kidney, where they receive a limited number of primary tubules, and thence pass, without receiving any others, in a direct vertical direction into the medullary substance. Here, in uniting with others of their own kind, they form larger tubules from which, by successive union, the ductus papillares finally result.

The initial or convoluted portion of the uriniferous tubule is lined by an epithelium consisting of cells of a turbid appearance, and of a slightly conical form, resting with their bases upon the interior surface of the basement-membrane of the tube, and presenting their smallest surfaces (the apices) to the lumen of the tube. In the narrow descending limb, to some distance beyond the loop of Henle, the epithelium consists of smaller cells arranged in the form of a pavement, their nuclei projecting into the interior of the tube. In the ascending limb, the cells assume a somewhat squamous arrangement up to the intermediate canal, which is lined by the same epithelium as found in the convoluted portion of the tubule. The epithelium found in the collecting tubules, and in those larger ones formed by the latter, consists of slightly conical cells, presenting their smallest surfaces to the lumen of the tube.

In the same manner as the kidney is composed of a certain number of conical or pyramidal bodies, these, on their part, consist of a considerable number of primitive cones formed by bundles of collecting tubules, together with the primary tubules they receive. These bundles may be regarded as separate lobules of the gland, for all the collecting tubules of one bundle are received by one and the same of the larger tubules, by which, finally, the ductus papillares are formed. And it is by these separate bundles of collecting tubules that the peculiar striated appearance, observed on the cut surface of the organ, is produced, the striæ being formed by the bundles and their interspaces. In the latter, the blood vessels are lodged.

The distribution of the blood vessels in the kidney is rather peculiar. The smallest branches, resulting from a successive division of the renal artery, proceed at once to the inner border of the cortical substance. Here they give rise to two sets of minute vessels—the *inter-lobular* (arteriæ interlobulares) and the *straight* arteries (arteriæ rectæ), which proceed in opposite directions. The interlobular arteries enter the cortical substance, and, lodged in the interspaces of the primitive cones, pursue their course toward the surface of the kidney, while, at the same time, they send minute arterial twigs (vasa afferentia) to the Malpighian bodies. Each of these arterioles penetrates the capsule of one of the latter, and then divides into a limited number of capillary vessels, which, after having formed a number of loops among themselves, reunite to form again another minute vessel (vasa efferentia), which leaves the capsule near the place at which the afferent vessel had entered. The conglomeration of vessels thus formed in the interior of the capsule represents the so-called "glomerulus." After having left the glomerulus, the efferent vessel terminates in the true capillary network surrounding the uriniferous tubules of the cortical substance, and giving rise to the radicles of interlobular veins.

The straight arteries, arising by a short trunk from the same renal branch as the interlobular, enter the medullary substance, in which, lodged in the interspaces of the primitive bundles of tubules, they give rise, successively, to small bundles of very fine straight vessels, the subordinate branches of which terminate in the regular capillary network, surrounding the tubules of the medullary substance, and consisting of long meshes. The vasa efferentia of the glomeruli near the border of the cortical substance, also, join this network of capillaries, in which the venous radicles of the straight veins arise.

Before dismissing the anatomical construction of the renal parenchyma, it may be further remarked that the glomerulus has no direct connection with the interior surface of the capsule, being only suspended in the cavity of the latter by its root, formed by the afferent and efferent vessel; neither does the liquid contained in the cavity directly touch the walls of the capillary vessels, they being covered by a layer of delicate cells, the nuclei of which are conspicuously distributed over the entire glomerulus.

While in the parenchyma of the liver the degenerative process, as we have seen, chiefly consists in an infiltration of fatty matters, derived from the blood and absorbed by the hepatic cells, we find it represented in the kidney by a true degeneration of the protoplasm of the epithelial cells lining the uriniferous tubules. The pathological process in the kidney appears to be initiated, as in the liver, by a general hyperæmia of the organ, the traces of which may still be detected by the microscopical examination. But, as in most fatal cases of yellow fever the congestion has, at the time of death, become greatly diminished by the ensuing degeneration, it is not often, when the autopsy is made, that the kidneys present a highly congested condition. Nevertheless, I have met, in former epidemics, with a limited number of cases, in which the kidneys presented the reddish-blue color of congestion to a considerable degree. And even in those kidneys in which the degenerative changes have already become perceptible to a microscopical examination, some portions of the organ may still be found in a state of hyperæmia. The traces of the congestive stage of the kidneys are best studied in thin sections. The microscopical examination of these sections shows that, in most cases, the congestion was at the time of death confined to the straight and interlobular veins, which are still found filled with blood corpuscles. However, not unfrequently a number of the capillary vessels, especially those along the border of the medullary substance (Grenzschicht of *Ludwig*), forming oblong meshes, are also found filled with blood. And, moreover, in some instances, I have even observed the minute vessels of a number of glomeruli in the same condition, indicating that the congestion must have extended throughout the capillary network of the cortical substance; and, in considering the contraction of the minute arteries, very probably taken place at the time of death, it may be presumed that, even in those cases in which only the minute veins were found filled, the congestion had likewise existed in the capillary network before the fatal issue took place. The presence of hæmoglobin, which in a number of cases may be demonstrated in the epithelium of the uriniferous tubules, moreover, corroborates the latter supposition.

The degenerative process, taking place in the renal parenchyma during the course of yellow fever, is in some respects peculiar, and appears not to resemble, in all points, the fatty degeneration of the kidney observed in connection with parenchymatous nephritis. The chief part of the process rather consists in a gradual breaking down and dissolution of the epithelial cells of an indefinite number of uriniferous tubules, terminating in most instances in a fatty metamorphosis of the remains. As a result of the degeneration, numerous so-called albuminous cylinders, and other infarctions, differing in composition and form, are formed in the interior of a considerable number of uriniferous tubules. These urinary infarctions, met with in certain organic affections of the kidney, have, since their discovery, always attracted the attention of pathologists, and as the probable origin and mode of formation seems to be still an open question, I shall endeavor to render a true account of their appearance in the yellow fever kidney, and also of their relationship to the degenerated epithelial cells. In doing so, I shall discuss the most prominent points of the question with special reference to their bearing upon the pathology of yellow fever. But, in order to facilitate the description of the condition and probable nature of the degenerated cells, and of the infarctions, I should make some preceding remarks upon the advantages derived from staining the thin sections of kidney with carmine, a valuable accessory in these studies, for the purpose of discriminating correctly between the different degrees of degeneration in which the cells may be affected, and also for determining the particular nature of the different forms of infarctions.

Knowing, namely, that the absorptive power of an organic cell is proportionate to the normal condition of its protoplasm, the extent or degree of the degenerative process, going on in the latter, may be determined by the degree of the coloring. The epithelial cells of the uriniferous tubules, therefore, will be perfectly colored as long as they are in their normal condition, but as soon as their protoplasm commences to degenerate, their absorptive power diminishes, and they appear only faintly colored; or, if the process of degeneration has farther advanced, the protoplasm remains colorless, while the nucleus may still be colored, or, if likewise degenerated, remain uncolored. The fatty matters resulting from the metamorphosis, remain, of course, uncolored, and appear yellowish when seen under the microscope. The so-called albuminous cylinders possess a considerable power of absorption, and appear highly colored. If hæmoglobin is present in the cells, they assume, in proportion to its quantity a more or less brown color; in relation to this body, especially, the carmine is preferable to other colors.

The infarctions formed in the uriniferous tubules during the course of yellow fever, differ somewhat from each other in their origin and formation. A number of them represent albuminous cylinders, while others consist of the remains of degenerated epithelial cells, or may even be composed of both. They are met with in all portions of the uriniferous tubules, and in correspondence with the diameter of these canals, vary in thickness. The largest are found in the convoluted portions, the so-called tubuli contorti, and in the intermediate canals (Schaltstuecke of *Schweigger-Seidel*), which they frequently fill up throughout their entire length. In the ascending and descending limbs and in the collecting tubules, they only exceptionally attain a great length, being generally short in extent. The relative number in which they are found differs considerably in different kidneys, and is proportionate to the extent of the pathological condition of the organ, which, as has been remarked before, greatly differs in different cases. Neither do the particular forms of infarctions stand in any fixed relation to the extent of the destructive changes in the kidney, for, while a considerable number of the simple albuminous cylinders may be met with in one case in which these changes exist to a great extent, they may hardly be observed in another case, in which only a comparatively small number of uriniferous tubules may be affected by the degeneration, or be obstructed by another form of infarction. In the same way does the degree of the degenerative process not always correspond to the extent in the organ, for there are cases met with in which few, or even no albuminous cylinders are observed, and in which the epithelium of only a small number of uriniferous tubules are found to undergo degeneration, but in which the latter process has nevertheless arrived at the fatty metamorphosis,—while there are other cases again, in which the formation of fat is hardly observed. To explain this discrepancy in the extent or degree of the pathological changes in the yellow fever kidneys, the comparative length of time preceding the fatal issue, and also the severity of the attack, must be considered; for, in those cases of a rapid course, and in which the patient succumbs in a few days from the effects of the cerebral disturbances, the kidneys may be found affected to only a small extent, while in other, more protracted cases, more time is afforded to the pathological process to extend farther throughout the organ.

Before proceeding to the description of the infarctions, a previous examination of the particular condition of the epithelium of the uriniferous tubules, which not only furnishes the material to the greater number of the obstructions, but, moreover is probably otherwise instrumental in their formation, may not be considered out of place. And, for this purpose, I shall refer in my description to one of those cases, in which the pathological changes observed are decidedly pronounced.

In examining a very thin section of kidney of such a case (Fig. 7), perfectly stained with carmine, it is found that the epithelium in the tubules has considerably diminished in its normal thickness (*b*). This observation is made as well on the entire tubule as on the transverse section of it. In the convoluted portions of the tubules, especially those surfaces of the cells directed toward the lumen, and which in the normal condition are conical, are now observed to be flat, causing the cells to appear rectangular in form. The lumen of such a tubule, as it is seen in a transverse section, therefore, will be found much larger than it is in the normal condition, though the epithelium may be intact; this is owing to the cells having lost their turgescent and conical appearance. In many cells, especially when viewed in profile, the nucleus appears more deeply colored than the protoplasm of the cell, while in others the color is more evenly diffused throughout the whole. In the greater number of tubules, the epithelium appears intact, but there are many others in which it is detached from the basement-membrane, leaving an interspace between it and the latter. In a number of other tubules, portions of the epithelium are observed to be completely separated from the basement-membrane, and to project into the lumen (Figs. 7, 8 and 9), or, its cells may be seen separating from each other, singly, or in small shreds. In some tubules, finally, the entire epithelium appears broken up, and with the exception of a small number of very faintly colored, or colorless cells, still adhering to the basement-membrane, either in small groups or singly, the whole tubule appears empty.

The cells of the epithelium, however, while undergoing this process of atrophy and disintegration, show as yet no traces of fatty metamorphosis. Most of those, still in contact with the basement-membrane, and, in severe cases, even forming the greater portion in the section, have absorbed carmine, especially their nuclei. But as soon as they are detached from this membrane, their power of absorption diminishes, and they appear but very faintly colored; or even, while the nucleus may still present a feeble carmine tint, the protoplasm of the cell has remained uncolored. It is not until they separate from each other that their degeneration and disintegration is really perceptible. The destructive process appears to consist in a dissolution or melting down of the hyaline portion of the protoplasm, liberating the granules, which, together with the hyaline substance, finally undergo a fatty metamorphosis (Figs. 11 to 15). At any rate, the granular remains of these cells do, at first, not always present a fatty appearance, though, soon after, the dark contours and the refractive property of the minute granules give sufficient evidence of their fatty nature. Very often, larger or smaller fragments of cells are observed to form a part of these remains, indicating again the grouped arrangement of the granules of the protoplasm, to which I referred in connection with the hepatic cells. In consequence of the breaking down of the protoplasm of the cells, the nuclei are set free, and traveling along with the general mass, they form a part of the resulting infarctions, in which they are almost always observed, either singly, or in clusters. They are then distinguished by being higher colored than the rest of the accumulation.

It is a remarkable fact that, during this process of fatty metamorphosis, taking place in the epithelium of the uriniferous tubules, it is quite rare that larger fat-globules are met with. I have examined a great number of sections for this special object, and have also found larger fat-globules in some of the tubules, especially in those near the capsule; but the number of these instances is so limited that their occurrence may rather be regarded as an exception to the rule. The comparative rarity, or absence, of larger fat-globules may be explained by the constant flow of some urine, even through these obstructed tubules, interfering with their formation. This supposition is corroborated by the streaked, thread-like appearance of the fat as it is observed in the interior of the tubules, particularly directly below the infarctions. Here it appears in long streaks of a yellowish color (Fig. 14), forming quite a contrast to the carmine coloring of the cells. Minute fatty granules, distinguished by their dark contours and high refraction, together with other cellular debris, are observed with these streaks.

In consequence of the degeneration and disintegration of the epithelium, many portions of the tubules are met with entirely denuded of their epithelial lining (Figs. 7 and 8, *d*). Although in the convoluted portions this condition is also observed, it is, nevertheless, more frequently found in the smaller tubules; and it is remarkable that not only single tubules are met with stripped in this manner of their epithelium, but generally a whole group or bundle, including descending, ascending, and collecting tubules. The only explanation which I can find for this phenomenon is that these groups represent certain arterial districts. But, even in these empty tubules, epithelial cells, singly or in patches, together with accumulations of granules and nuclei, may frequently be observed. In most instances, perhaps, the empty tubules have preserved their normal caliber, though not unfrequently groups are met with in a collapsed condition, their basement-membrane presenting faint outlines.

Besides the condition of atrophy and degeneration just described, in which the epithelial cells of the uriniferous tubules are found in yellow fever, there is another condition, indicated by a peculiar appearance of a considerable number of cells, and which seems to precede that of atrophy and degeneration. The special description and consideration of this condition, however, I must postpone until I have described the appearance and the nature of the infarctions.

These infarctions, besides differing from each other in composition, are met with in the uriniferous tubules in different conditions. The so-called albuminous cylinders, when examined in an uncolored section, appear under the microscope finely granular, amorphous, faintly glistening, and of a yellowish color. They are, however, studied to a greater advantage in sections stained with carmine, in which, with the exception of the color, they present, especially in transverse sections of the tubules, the same characters. Possessing, as already mentioned, the power of absorbing coloring materials in a very high degree, they appear in such sections very highly colored, and exhibit the carmine tint with a greater brilliancy and intensity than the normal protoplasm of the cells; therefore, when they are met with in their pure condition, that is, unmixed with epithelial fragments and remains (Fig. 7, *a*), they are easily recognized by their brilliant and even coloring. They generally fill up the whole lumen of the tubule, and mostly terminate in tapering, rounded-off extremities. In most instances, perhaps, they are still surrounded by the epithelium, though not unfrequently they are met with in denuded portions of the tubules, and in direct contact with the basement-membrane. When covered by epithelium, the outlines of the cells may still be recognized, though the whole cylinder appears somewhat darker. Many of these cylinders are found in a broken condition, either simply fractured across, or into a number of greater or smaller fragments of rectangular or polyhedral forms. It is difficult to determine the cause of these fractures, though it may be safely presumed that they occurred by the instrumentality of the knife during the cutting of the sections. In the study of these sections, therefore, the investigator should take this circumstance into account, in order to guard against false conclusions, which he otherwise might draw from his observation. There are, nevertheless, specimens of these broken cylinders here and there met with, of which the fragments appear to have been removed from each other after the occurrence of the fracture, while their angular borders appear slightly rounded, as if washed away by the passage of the urine. Whatever the cause of these fractures may be, they, themselves, at least, give evidence of the brittle nature of these cylinders, which, accordingly, bear resemblance to the so-called "waxy cylinders," frequently met with in interstitial nephritis. A number of these cylinders are observed, which appear distinctly granular in their composition, the very minute granules exhibiting dark contours. Not unfrequently the lower end of the cylinder is in a state of dissolution, when the individual granules may be distinctly seen. And if the very border of the disintegrating extremity is closely examined, some of the granules may almost always be observed exhibiting their fatty nature by their refractive property and dark contours.

Perhaps the greater portion of these cylinders is found to contain epithelial remains, consisting of granules, nuclei, and even entire cells, or their fragments (Fig. 8, *a*). Mostly, their morphological elements of the epithelium are irregularly mixed up into a shapeless mass, but very frequently cylinders are observed containing, besides the granules, a whole cluster of nuclei, or even entire cells. If these fragmentary remains of the epithelium have, as yet, not undergone the fatty metamorphosis, they appear more intensely colored than the original substance of the cylinder itself, and exhibit very dark contours and a peculiar luster. But not unfrequently instances are met with in which a portion, or the whole, of these epithelial remains have already undergone this metamorphosis, and accordingly appear lighter, or even yellowish, but also with dark contours, especially the granules (Fig. 15, *a*). The degenerated epithelial elements, not enclosed in the cylinder, of course, exhibit distinctly the fatty appearance, as before described. As regards the relative proportion of the albuminous substance of the cylinders with the epithelial remains, it, of course, must differ in every combination of cylinder formed, and be subject to much variation. It may be presumed that the epithelial elements have accumulated in the lumen of the tubule previous to their saturation by the albuminous substance, to which the intense coloring must be attributed. Judging from the tapering form of the extremities of these mixed cylinders, and also from the irregular outlines observed on some of them, they appear to be prone to a gradual liquefaction.

A third kind of infarctions met with in the uriniferous tubules, in yellow fever, are those solely composed of the disintegrated and degenerated epithelial elements. They consist of granular masses of irregular forms, derived from the degenerated cells, and mostly enclosing free nuclei and cells, or their fragments; sometimes, however, clusters of entire nuclei and cells are observed (Fig. 8, *e*). The size or extent of these accumulations is very variable, but it may be stated that, as long as they remain free from being saturated by the albuminous liquid, they never assume the same dimensions as the albuminous cylinders above described, though they are met with in the same localities as the latter. Neither do they appear to become very easily stationary in one particular place of the tubule; on the contrary, being of a softer consistence, and more yielding, and, furthermore, undergoing easily fatty metamorphosis, they are carried along by the pressure of the urine. These epithelial accumulations are frequently observed in a state of fatty degeneration, the whole mass appearing yellow, or, sometimes, yellowish mixed with brown; in the latter instances the brown color represents hæmoglobin, previously absorbed by the protoplasm of the cells.

Having thus far described the degenerated condition of the epithelium, lining the uriniferous tubules, and the peculiarities of the different kinds of infarctions, formed in the latter during the comparatively brief course of yellow fever, I shall now consider the probable origin and mode of formation of these infarctions, and, moreover, briefly review the existing theories on the subject.

The old theory of the mode of formation of the so-called albuminous cylinders referred the process to a transudation of the albumen of the blood through the walls and the covering cells of the minute vessels of the glomeruli into the uriniferous tubules, in the interior of which the cylinders were formed by a precipitation of the albumen from the urine This theory is entertained by perhaps the majority of pathologists, though the other, more recent, according to which the cylinders represent a product of secretion from the epithelial cells, or are even the result of a transformation of these cells themselves, is also supported by a number of distinguished authorities. To myself, the old theory hitherto appeared sufficiently plausible to explain the whole phenomenon, though I must confess that an observation which I made during these studies on a considerable number of epithelial cells, to be described hereafter, has induced me to view the more recent theory in a different light. Let us, therefore, examine both sides of the question.

As the old theory is sufficiently understood to pass without further explanation, I shall, in support of it, only cite the views of *Runeberg*,* one of the more recent investigators into the pathogenetic condition of albuminuria. They are as follows: "The transudation of serum albumen always takes place in the glomeruli. It is conditioned by an increased permeability of the walls of the looped vessels of the glomeruli and of the epithelium covering them. In consequence, the particles of albumen suspended in the serum of the blood, which under normal conditions

* *Virchow u. Hirsch*, Jahresbericht etc., für das Jahr 1878, Vol. I, p. 219.

are unable to pass through the membranes of the glomeruli, may now in part be filtered with the remaining constituents of the urine. This increase of permeability is, in otherwise healthy kidneys, already caused by a considerable diminution of the difference between the pressure of the blood inside the glomeruli and the counter pressure existing in the uriniferous tubules. The accidental or transitory albuminuria is, therefore, conditioned by a considerable augmentation of the presence of the blood in the glomeruli, or by a diminution of the pressure in the tubules, or by both of these circumstances. In the permanent albuminuria, however, increased permeability of the filtering membrane is conditioned by an inflammatory or degenerative process affecting the vessels of the glomeruli; but, even here, the permeability is perceptibly influenced by the relative amount of pressure, and, in the same direction, also the amount of albumen in the urine, as explained before. A part of the albuminous substances, as the egg albumen and the hæmoglobin, are in a higher degree filterable than the serum albumen. As soon, therefore, as these substances are, in any way, mixed with the serum of the blood, they at once, even under normal conditions of the pressure of the blood, filter into the urine like the soluble salts."

The more recent theory, according to which the cylinders represent a product of secretion from the epithelial cells of the uriniferous tubules, or result from a transformation of these cells themselves, has been advocated by *Oedmansson*, *Axel Key*, *Oertel*, *Rovida*, *Senator*, *Heynsius*, *Bayer*, *Birch-Hirschfeld*, *Aufrecht*, and probably by a number of other pathologists. I shall cite the views of some of these authors. Thus, *Rovida** who subjected the urinary cylinders to an extensive chemical examination distinguishes three particular kinds, the *colorless*, the *yellow*, and the *epithelial*. As regards the chemical nature of the colorless, and the yellow or waxy cylinders, he says, that they do not represent albumin, or an albuminate, nor one of the known bodies derived from albumen, although they chemically resemble the latter substances.

* l. c.—für das Jahr 1872, Vol. I, p. 206.

He states a case of diffused nephritis without contraction or amyloid degeneration of the kidney, in which, beside colorless and yellow cylinders, numerous yellowish minute scales, exhibiting a similar luster as the latter, had been observed in the urine during the life of the patient. The same elements were met with in the fresh kidney, when microscopically examined after death. After the organ had been hardened in Mueller's fluid and in alcohol, the epithelium of the convoluted tubules appeared turbid and granular, so that the nuclei could scarcely be distinguished; the lumen was filled up, partly with homogeneous, partly with slightly granular globules of the same color and refractive as the yellow cylinders. In many uriniferous tubules, spherical drops of yellow color were observed, especially in transverse sections, to project from the epithelium; they were also found, here and there, in the lumen, partially collected into irregular polyhedral figures. In other places they formed an almost spherical contour, blending toward the center with a firmer, more compact mass, which, filling up more or less perfectly the lumen of the uriniferous tubule, represented a yellow urinary cylinder. From this observation, Rovida concludes, that the yellow cylinders, also, are products of secretion from the epithelium of the uriniferous tubules, the same as had been previously shown by Oedmansson, Key, and Oertel in connection with the colorless cylinders. But, finding in the same kidney, in some places, especially in the descending and ascending tubules and in the loop of Henle, the epithelium yellowish and more refracting, he agrees with Key, in admitting that the cylinders may also be formed by a fusion of transformed cells.

*Senator*,* who also made numerous investigations upon the albuminous bodies in the urine, regards albuminuria as depending upon an abnormal circulation. The increase of tension which the vessels of the glomeruli experience during a general venous congestion, he thinks, is inferior in degree to that of all other capillaries, while at the same time the secretion is diminished by an accumulation of the secreted matters in the uriniferous tubules. In addition, a diminution of the arterial pressure,

* l. c.—für das Jahr 1874, Vol. I, p. 342.

as usually observed during venous congestion, occurs. Therefore, the albumen cannot be regarded as a product of filtration from the glomeruli, but is probably derived from the interstitial vessels bearing high pressure. In pure cases of amyloid degeneration, the urine voided from the Malpighian canals must be regarded as a mixture of a serous, non-inflammatory transudation, pressed through the glomeruli. The changes, occuring in the different forms of diffused, interstitial and parenchymatous nephritis in the urine, are depending upon the effects of the changed afflux and reflux of the blood in the vessels of the glomeruli, and in the interstitial vessels, and from those caused by the accumulation of secreted matters in the uriniferous tubules. The albuminous cylinders Senator regards as a product of a disturbance of nutrition of the epithelium.

*Heynsius*,* who closely investigated the same subject, agrees with Senator in the following points : namely, that the albuminous bodies met in the urine need not resemble those of the plasma of the blood in all points ; that the epithelium of the kidney undoubtedly possesses some influence over the secretion in general, and over that of the albumen in particular; and that the cylinders are not formed by transudation from the plasma of the blood, but are rather derived from the protoplasm of the epithelium.

The cause of albuminuria, he thinks, may be found not only in an increased venous pressure, but also in a diseased condition of the epithelium of the uriniferous tubules, in consequence of which the quantity of albumen, transuding through the vessels in the normal condition, is not consumed for the nutrition of the epithelium. The destruction of the latter, however, causes a diminution of the acid reaction, followed by an increase in the quantity of albumen.

*Birch-Hirschfeld* † adopts the view of Key, according to which the hyaline cylinders are probably formed by a secretion of the epithelium, while the granular cylinders are formed by a fusion of the degenerated epithelial cells.

*Aufrecht* ‡ found that in the rabbit, after ligation of the ureter on one side, a granular turbidity of the epithelial cells in the

* l. c. p. 243.
† *Birch-Hirschfeld.*—"Lehrbuch der Pathologischen Anatomy," 1877, p. 1038.
‡ *Virchow u. Hirsch*, Jahresbericht, etc., f. d. Jahr 1878, Vol. I, p. 222.

cortical substance of the kidney of the same side, associated with the formation of cylinders takes place, followed—about six days after the ligation—by a proliferation of cells in the interstices of the uriniferous tubules. He agrees with Oedmansson, Axel Key, Senator, and others, that the cylinders are formed by the irritated epithelial cells, and represent a secretion, which, in several instances, he has seen projecting from the cells.

Having thus briefly reviewed the leading theories on the formation of the albuminous cylinders, and, moreover, cited the particular views of some of the investigators, I shall now proceed to state my own observations on the subject. In the preceding description of the condition of the epithelium of the uriniferous tubules, the degree of degeneration in which the cells were found, was, as will be remembered, determined by the capacity of absorption which they showed for coloring matters. In accordance with this test, those least, or not at all, degenerated, exhibited a perfect staining, while others were more imperfectly colored, or had remained entirely colorless. These cells, moreover, without reference to the quantity of carmine absorbed, did not present any lustrous or glistening appearance, nor that intense color exhibited by the urinary infarctions. There are, however, a considerable number of others, as yet not mentioned, met with in all sections of yellow fever kidneys, which do present *the same intense color and glistening aspect as the infarctions.* They are particularly well exhibited in transverse sections of the uriniferous tubules in which the epithelium appears in the form of a ring, though they are also distinguished in others, or even in the entire tubule through the basement-membrane. In many instances, only a certain number of the cells, forming the ring, present this peculiar appearance (Fig. 9), though in others, all the cells of the epithelial ring appear affected (Fig. 10).

These deeply colored and glistening cells are not confined to any particular locality, but are met with in all portions of the tubules. Neither are they confined to particular cases, for I observed them in all the sections I examined, and without reference to the greater or lesser extent of the destructive changes in the parenchyma of the kidney. They were rather more frequently observed in the milder cases, in which the process of degeneration was of a limited extent. In such cases, I also observed this characteristic appearance on a number of cells, lining some of the collecting tubules, and which showed no signs of atrophy or degeneration besides (Fig. 10, *a*), but had preserved their normal size and form. In some of these sections, no cylinders of notable size were found; those met with were contained in the smaller tubules and very short. In these instances, however, a number of transverse sections of the smaller tubules were also observed, in which not only the epithelial ring exhibited the deeply stained and glistening appearance, but in which the lumen was moreover filled with a cylinder (Fig. 10 *b*), presenting the appearance of being blended with the ring, such as was observed by Rovida in the uncolored specimen.

It will be obvious that these observations strongly corroborate the more recent theory, according to which the albuminous cylinders are a product of secretion from the epithelial cells of the uriniferous tubules, or may even be formed by a fusion of the degenerated cells themselves. Some additional remarks, however, will be required to render the probable correctness of this theory more comprehensible. It has already been mentioned that in many transverse sections of the albuminoid cylinders, unmixed with epithelial remains, the epithelium is found perfectly intact, and, though decreased in its normal thickness, nevertheless appears evenly colored, showing the absence of fatty metamorphosis. This fact has been advanced by *Weissgerber* and *Perls*,* for the purpose of showing the fallacy of the theory in question, but loses its significance if explained in another way. These investigators probably supposed that if the cylinders were a product of secretion from these cells, the latter should still contain a portion of this product, and accordingly present the same yellowish and glistening aspect as the cylinders. This condition of things, it is true, may be conjectured, and I presume does, in some instances, exist, and may sometimes also be observed, though its actual observation is by no means essential; for, having observed the deep color and glistening appearance on the cells of collecting tubules—in some instances on the entire epithelial ring—which had preserved their normal turgescent form, I am

* l. c.—fuer das, 1876.—Vol. I, p. 263.

inclined to regard this abnormal secretion as the initial stage of the degenerative process, followed, directly after the discharge of the secreted product, by the atrophy of the cells, before mentioned. It is, therefore, not essential that the cells surrounding a cylinder, representing the discharged product of their secretion, should in all cases still present the optical characters of this product; on the contrary, after the complete discharge of the secretion, they may not only resume their former appearance, but at the same time appear atrophied by the loss of material. But even if this supposition were wrong, there remain other probabilities of the cylinders being in truth a product of secretion; for it is not essential that this product should have come from those particular cells by which it is surrounded. On the contrary, it is more likely that it has been secreted from cells situated higher up, and, being at the time of secretion in a semi-fluid condition, has descended until arrested by the ensuing coagulation at another part of the tubule, in which the epithelial cells have not been similarly affected.

In some instances, as has been mentioned, the epithelium, generally surrounding the cylinder or infarction, has entirely disappeared, the latter being in direct contact with the basement-membrane. In these cases it may be presumed that these infarctions, consisting of epithelial remains saturated with the albuminoid matter, were probably formed in the place where they are found, while purely albuminoid cylinders are derived from cells situated higher up in the tubules.

It has already been noted that, in some instances, the minute vessels of the glomeruli were found filled with blood-corpuscles, showing that the congestion had extended throughout the whole capillary network of the interlobular vessels; generally, however, the vessels of the glomeruli were empty, and appeared normally colored; in a few instances they even appeared contracted, and the glomerulus diminished in size. In examining a considerable number of sections for the special purpose of discovering the peculiar condition of the epithelial cells above described, also on the layer of cells covering the vessels of the glomeruli, I only succeeded in observing in some parts of a few glomeruli the deep coloring and glistening appearance; besides this, I also met with the segments of a few capsules, upon the inner surface of which an exudate, moderately colored by the carmine, could be detected. Another observation, however, quite as interesting, consisted in the presence of fat-globules, such as might be derived from a fatty infiltration, in a number of the cells of the covering epithelial layer of many glomeruli. These observations show that these cells also are prone to be affected in the same manner as those lining the uriniferous tubules.

As in the liver and stomach, so in the kidney, extravasations of hæmoglobin take place from the capillary vessels, to be absorbed by the epithelial cells of the uriniferous tubules. Almost in every section of yellow fever kidney, some traces of free hæmoglobin, recognized in carmine preparations by their peculiar brown color before mentioned, may be observed in any portions of the tubules. Sometimes the extravasation is observed in a considerable number of neighboring tubules. The pigmentation, however, is not confined to the formless hæmoglobin, for hæmatoidin in granular or crystaline form is also met with. Hæmatoidin crystals, especially, are frequently seen upon the inner surface of the epithelium, generally collected in small groups or masses of very minute crystals, though in one particular case I also observed numerous granules of hæmatoidin in the interior of a number of empty uriniferous tubules. The brown color which the epithelial cells assume by the absorption of the hæmoglobin, of course, differs in intensity in different localities, according to the quantity absorbed. These extravasations are not limited to kidneys extensively degenerated, but are equally observed in cases presenting only slight traces of the degenerative process.

As far as my experience extends, there is no product of inflammation found in the yellow fever kidney. Although I had, in every section examined, my attention directed to this point, I failed to discover any trace of such a product, either in the vicinity of the connective tissue capsule, or in the interstitial tissue. Therefore the destructive changes occurring during the course of yellow fever, in the parenchyma of the kidney, are the result of hyperæmia.

In conclusion, I may remark that no traces of bacteria, or other minute organisms, could be discovered in this organ. If such organisms were really present in the kidney, as has been erroneously stated, we should most probably find them in the minute blood vessels, or in the interior of the uriniferous tubules. But not a single specimen, or colony, have I met with in these localities in the very numerous sections which I have examined. And if ever these organisms are met with in these places, a close investigation will certainly show that they were developed along with the decomposition of the body, or of the kidney itself, after its removal from the former. In one exceptional case only, I observed upon a section, mounted in Canada balsam, one or two granular patches, representing colonies of minute micrococci. A change of the focus, however, showed that they were not contained in the preparation, but were simply resting upon its surface, where they had been developed after the section had been cut, an accident which probably occurred in the staining fluid, or while the sections were kept in a mixture of alcohol and water, previous to the process of mounting.

### THE SUPRA-RENAL BODIES.

These organs, of which the true function is, as yet, not known, are in fatal cases of yellow fever almost invariably found to have undergone certain pathological changes. To render a description of these changes more perspicuous, we may, as before in some other instances, briefly review their microscopical anatomy.

As in the kidney, so in the supra-renal bodies, a "cortical" and a medullary substance have been recognized—though here no secretory ducts exist, but both substances consist of minute blood vessels and cells, supported by a fine reticulated network of connective tissue. This network originates by certain septa, or processes, arising from the inner surface of the general capsule of the organ, whence it extends throughout the parenchyma, to be finally connected with the adventitia of the veins, which leave the organ through its hilus. The blood-vessels of the organ are very numerous, and represent branches of the aorta, and of the phrenic, cœliac, and renal arteries, which, after penetrating the capsule, subdivide into numerous minute branches, terminating in the capillaries of the cortical substance. Three distinct layers of cells are distinguished in this substance. The middle one of these layers truly represents the bulk of the cortical substance, while the outer and inner ones only appear as narrow borders. The inner layer blends with the adjoining medullary substance. The cells, which are polygonal or round in form, occupy the space between the septa in the form of larger or smaller groups or columns. In the outer and inner layer, these groups, in accordance with the smaller and rounder meshes of the reticulum, are small and of a roundish form, while in the middle layer, in which the meshes are larger and much longer, the groups of cells assume the forms of columns. The medullary substance consists of a reticulum of very delicate connective tissue, with small meshes, filled up by small groups of polygonal or stellated cells, containing large nuclei. The capillaries, differing in their caliber in the different localities of the parenchyma, generally follow the septa and columns of the reticulated connective tissue, and, accordingly, the form and size of their meshes correspond with those of the meshes of the reticulum. Their caliber is of a medium size in the cortical substance, but in reaching the medullary substance, in which they represent a network with small meshes, they considerably enlarge in diameter, and many of them even present dilatations. In the latter network, the venous radicles take their origin. The nerves in the supra-renal bodies are very numerous; they are derived from the semi-lunar ganglion, the renal plexus, and the phrenic and pneumogastric nerves, and, after having entered the organ, are chiefly distributed to the medullary substance, forming plexuses in connection with which ganglion-cells have also been observed. The pathological changes observed in the supra-renal bodies resemble those taking place in other organs already described, consisting in the infiltration of fat and hæmoglobin, derived from the blood, and preceded by hyperæmia of the organ. In most cases, therefore, the examination of thin microscopical sections shows the capillaries filled with blood-corpuscles, especially those larger ones extending through the medullary substance, though in a number of cases I also observed the others, particularly those of the outer layer of the cortical substance, congested. The fat-globules, resulting from the fatty infiltration, are quite large, and are found in the cells of both substances, though perhaps to a greater extent in the cortical.

The infiltration or extravasation of hæmoglobin absorbed by the cells is greater and more general here than has been observed in any other organ. Almost in every case examined, it involved the whole medullary substance, from which it extended into the inner and middle layer, sometimes even into portions of the outer layer of the cortical substance. The degree of this pigmental infiltration is sufficiently great to be always distinguished by its brown color in sections of fresh specimens; in some cases, even, it appears dark brown. In sections, stained with carmine, the infiltrated cells exhibit the characteristic brown color of the extravasated hæmoglobin, already referred to. Besides the fatty and pigmental infiltration met with in every specimen examined, I moreover observed, in some cases, that a process of atrophy of the parenchymatous cells, particularly those of the medullary substance, had also been going on. In some cases, even, the atrophy was associated with softening of this substance, which presented a dark brown color; the softening process had given rise to a cavity in the organ. The atrophy appeared to be chiefly confined to the protoplasm of the cells, the nuclei remaining unaffected.

For the sake of comparison, I prepared a number of sections of supra-renal bodies taken from several cases representing other diseases than yellow fever, such as of the heart, liver, kidneys, and lungs. In these sections, a trace of pigmental infiltration was detected, but too insignificant to be in any way compared with that observed in yellow fever, as it was entirely limited to a very narrow yellowish brown stripe, representing the inner border of the cortical substance.

Although a number of pathological conditions and structural changes, such as hypertrophy, atrophy, fatty and amyloid degeneration, suppurative inflammation, carcinoma, tubercle, cysts, and even extensive hæmorrhages in the interior of the organ, have been observed to occur in the supra-renal bodies, I have thus far seen no record of an infiltration of hæmoglobin into the parenchyma in such a marked degree as I have observed to take place in yellow fever,—a phenomenon to which some special signification regarding the normal function of these organs might be attached, if it was not simultaneously observed in the liver, stomach, and kidney. For this reason, it must be regarded, as in the other cases, depending upon the hyperæmic condition of the vessels, retarding the circulation of the blood through the latter. The fatty infiltration, also, is evidently due to the same general causes, from which it depends in the other organs already mentioned.

THE CEREBRO-SPINAL AXIS.

*The Pia Mater.*—In connection with the macroscopical examination of the organs after death, I stated that this membrane, as far as it extends over the brain, was almost invariably found in a state of hyperæmia, and that, in many cases, not only the veins, but also the arteries were found filled with blood. The microscopical examination reveals the same condition in the minute vessels, the arterioles, venules and capillaries. In examining, therefore, a small piece of pia mater, carefully removed from the cortex cerebri, these vessels, with only a few exceptions, are, in most instances, found more or less filled with blood corpuscles; in some of them, even, the corpuscles are crowded to such an extent as to have lost their original form by the mutual pressure which they had exerted upon each other. In removing the pia mater, the minute vessels entering the substance of the brain are, of course, torn, causing the blood corpuscles which they contain to escape. The stumps of these vessels, appearing like villi, and attached to the inner surface of the membrane, are usually found empty. Notwithstanding the intense hyperæmia which appears to have existed during life in many portions of the pia mater of the brain, I have, with one single exception, failed to detect any exudation cells in the vicinity of the vessels, or any increase in the connective tissue cells of the membrane, which might have indicated the pre-existence of inflammation,—a fact, which clearly shows that the cerebral phenomena observed at the bedside, are the results of a simple though severe hyperæmia. In the one exceptional case, the connective tissue cells had just commenced to undergo a division, resulting in a small group of three or four cells, or only nuclei, in the place of the old ones (Fig. 17). In some cases, I have observed that the walls of a number of arterioles, especially of those which are empty, or containing but few blood corpuscles, presented a corrugated or wrinkled appearance. As the wrinkles were confined to the adventitia, they appear to have been caused by a contraction of the delicate muscular fibers, encircling these small vessels. If this be the true cause, the contraction must have occurred before death, or during the agony of death itself. But there is another pathological condition, which, in fully one half of the cases examined I observed to have taken place in these minute vessels, both arterioles and venules, and which consisted in a fatty degeneration of their nuclei, especially of those belonging to the adventitia. In most of these instances, the nuclei have disappeared, leaving a group of smaller or larger fat globules in their places. In other instances, an increase in the mere trace of protoplasm, found in connection with the nucleus in its normal condition had taken place, causing a thickening of the wall of the minute vessel, and giving rise to a capillary aneurism, or, by its proneness to degeneration, even to a final rupture followed by hæmorrhage. The small hæmorrhagic effusions, observed in some of these cases, may have been owing to this cause.

The arachnoid membrane, as before stated, is very frequently found not only opaque, but also thickened. This condition is caused by an exudation of a finely granular matter into the subarachnoid space, filling up, at the same time, the meshes of the connective tissue of the pia mater. The granules of which this substance is composed, are quite distinct, but pale, and measure about $\frac{1}{1200}$ mm. in diameter. In the pia mater of some cases, and mostly in the close vicinity of a vessel, or associated with a minute hæmorrhagic effusion, smaller or larger brownish looking granular masses of irregular form, are here and there observed. The granules of these masses are identical with those of the exudate before mentioned, though they appear more distinct, which is probably owing to the presence of free hæmoglobin, imparting the color to the mass. A number of similar, but colorless granular accumulations are moreover observed in the spaces between the vessels. It is quite probable that these accumulations represent minute portions of granular substance from the upper stratum of the cortex cerebri.

The hyperæmia of the pia mater is not confined to those portions of the membrane covering the cerebrum and the cerebellum, but, moreover, extends over the pons varolii and medulla oblongata, frequently even as far as the cervical enlargement of the spinal marrow. In many cases, the congestion of the vessels of the pons and medulla is greater than that of the central vessels.

While in the cervical and dorsal regions, the pia mater of the spinal marrow is generally found free from congestion, its vessels are almost always found filled with blood corpuscles in the lumbar region. This fact can be sufficiently demonstrated in thin sections of spinal marrow from this region, in which the minute vessels of the membrane, with those that enter the spinal marrow, are observed filled with blood corpuscles.

*The Substance of the Brain.*—As it may well be asserted, that in yellow fever the safety of the patient almost entirely depends during the febrile stage, and after, upon the particular condition of the nervous system, especially the brain, an exact knowledge of the pathological changes taking place in this organ, are most important. And, judging from the traces of intense hyperæmia, met with in almost all fatal cases in the pia mater, as above described, we may well expect to find a similar condition in the substance of the brain. The pathological condition of this organ may be studied to the greatest advantage in large thin sections, passing through the whole extent of its different parts. The examination of a very great number of such sections revealed to me that, in almost every case examined, the hyperæmia extended from the pia mater *throughout* the substance of every portion of the brain. Not only the arterioles and venules were found filled with blood corpuscles, but also the capillaries. In most instances, the blood corpuscles are crowded in the arterioles and venules, and to such a degree as to have lost their normal form by their mutual pressure. In the pons varolii and medulla oblongata particularly, the vessels are invariably found filled with blood corpuscles, inducing me to regard these parts as special seats of the congestion. In some cases, I have even found opacity of the pia mater covering these localities. In those cases in which there was fatty degeneration of the arterioles and venules of the pia mater, this process is observed to have extended to the same minute vessels of the brain substance. In some cases, even, I observed traces of commencing degeneration in a considerable number of ganglion cells, especially on those of the cortex cerebri, but also in the medulla oblongata. While in a normal ganglion-cell, namely, the double contour of the nucleus, and the outlines of its granules and those of the protoplasm of the body, generally seem distinct, they here appeared indistinct, and the whole ganglion cell presented a fatty luster. This condition is best observed in uncolored sections, examined in water or glycerine. In one case, I observed that though the outlines of these anatomical constituents of the ganglionic bodies of the cortex cerebri had been well preserved, their protoplasm had undergone atrophy, reducing their size. Notwithstanding the occurrence of extravasations of hæmoglobin in every other organ examined, the pia mater not excepted, as I have shown before, the substance of the brain appears to be remarkably free from this pigmentary infiltration ; neither were in all the cases examined microscopically—about a dozen—capillary hæmorrhages met with.

As regards the *spinal marrow*, there are, aside from the congestion of the vessels of the pia mater above mentioned, no pathological changes observed in the nervous elements.

### THE GANGLIA OF THE SYMPATHETIC NERVOUS SYSTEM.

The microscopical examinations which I made on these ganglia, embraced a very considerable number of sections, made of the *first thoracic*—G. stellatum—and the *semi-lunar* ganglia of six cases. In the majority of these cases, the minute blood vessels of these ganglia were found filled with blood corpuscles. In two cases, the ganglionic bodies of the first thoracic, as well as of the semi-lunar ganglion, had most obviously undergone degeneration. In the greater part of these ganglion cells, the nuclei had entirely disappeared (Fig. 16), leaving no other trace but their nucleoli, which appeared very distinct ; in the rest, *very faint* outlines of the nuclei could be still observed. The bodies of all these ganglion cells presented an indistinct appearance, and were characterized by a peculiar fatty luster, even observed on specimens mounted in Canada balsam. As there were no fat globules observed in the places of the nuclei, it is difficult to account for their disappearance, unless it was caused by a process of atrophy; such as was observed to occur on the epithelial cells of the uriniferous tubules. In a third case, I observed an abnormal accumulation of pigment in a considerable number of ganglion cells of the semi-lunar ganglion. In the three remaining cases, nothing specially abnormal could be detected on the ganglion cells.*

The preceding description of the pathological changes taking place in various organs and tissues in the course of yellow fever, is based upon very numerous and careful examinations, made during former epidemics, but particularly upon the abundant material which I collected during the epidemic of 1878. In the pursuit of these studies, portions of the organs were examined in their fresh condition directly after the autopsy, while the rest were put into a simple solution of bi-chromate of potassa, or into Mueller's fluid. A few days later, after the tissue ha l been slightly hardened, the examinations were repeated upon thin sections made by the free hand; but after they had attained the proper consistency, larger and perfect sections were made by the aid of the microtome. These, finally, were carefully prepared, stained, and mounted in Canada balsam or glycerin, in order to serve for the more thorough final studies.

The amount of material which I have in this manner examined and studied is very great; it embraces the various organs of twenty-three cases out of the thirty autopsies which I made during the epidemic of 1878, and those of an additional most interesting case in 1879. I have made more than two thousand thin sections, ranging from the larger ones, passing throughout an entire hemisphere of the cerebrum to the smaller of the sympathetic ganglia, or the mucous membrane of the stomach. The

---

* In the "Report of the Havanna Yellow Fever Commission of the National Board of Health," it is stated that in the semi-lunar ganglion "connective tissue of new formation is met with to a greater extent than in the tissues heretofore described, but the nervous elements present no evidence of degeneration further than the cloudy swelling already described in other organs; while of the other portions of the nervous system which have not been examined microscopically, it may be said, that they present nothing abnormal to the naked eye."

The discrepancy existing between these statements and my own observations, I can only explain by presuming that the pathological expert of the commission took the very numerous nuclei, belonging to the capsules, and to the intricate extensive plexuses of sympathetic nervous fibrillæ connecting the ganglionic bodies, or to the bundles of sympathetic nerve fibers arising from them, for newly formed elements. As regards the report of the condition of the other portions of the nervous system, I could only infer that the type of yellow fever at Havanna must differ from that observed at New Orleans.

sections of the brain are especially numerous, comprising all parts of the organ, and representing a considerable number of cases. About one thousand of these sections, taken from different organs, I have mounted in Canada balsam or glycerin, a large portion of them being in the possession of medical friends in New York, Philadelphia and Chicago, while the rest remained in New Orleans.

A word concerning the mounting and examination of these or similar specimens may be advisable here, as their successful study much depends upon the medium in which they are examined. Thus, though the mounting in Canada balsam is the most substantial and beautiful for colored sections of tissue, there are nevertheless certain disadvantages connected with this medium, and depending upon its high degree of refractibility. This is the case with tissues that have suffered fatty infiltration or degeneration, in which the fat globules, by the too great amount of light passing through them, are rendered quite indistinct, and difficult of recognition. For this reason, it is better to examine and mount such specimens in a less refractive medium, such as glycerin. In examining stained tissues, mounted in balsam, they should not be too brilliantly illuminated, as too much light will render indistinct the image of structure, though it shows well the image of color. It is more advantageous, therefore, to use the plane mirror and a small diaphragmatic opening for the sections mounted in balsam, while the glycerin sections may be examined with the concave mirror. I need hardly mention that examinations of this kind should only be made with the very best first-class objectives, as an inferior objective does not show the details in their true light.

[TO BE CONTINUED.]

---

THE Minnesota State Medical Society will hold its annual meeting at St. Paul, on Tuesday, June 20, 1881. The profession is respectfully invited to add to the interest and value of the meeting by being present. ALEX. J. STONE, M.D., *President.*

